# The Mediation Role of Safety Attitude in the Impact of Resilience on the Safety Behavior of Coal Miners in China

**DOI:** 10.3390/ijerph192215164

**Published:** 2022-11-17

**Authors:** Yuanlong Li, Jingqi Gao, Chongyang Qian, Xiang Wu

**Affiliations:** 1 School of Engineering and Technology, China University of Geosciences (Beijing), Beijing 100083, China; 2 China Academy of Safety Science and Technology, Beijing 100012, China; 3 Institute of Urban Safety and Environmental Science, Beijing Academy of Science and Technology, Beijing 100054, China

**Keywords:** coal miners, resilience, safety behavior, safety attitude

## Abstract

Resilience can improve the adaptability of coal miners to high-hazard and high-stress environments. After facing setbacks or adversities, resilience can enable coal miners to recover from bad mental states and have an optimistic safety attitude and positive safety behaviors. However, how resilience affects safety behavior and the role of safety attitude in the relationship have not been clear. This study systematically reviewed previous research on resilience, safety attitude, and safety behavior. By recovering 639 valid questionnaires, the validity and reliability of the resilience scale, safety attitude scale, and safety behavior scale for coal miners were verified. Hierarchical regression analysis explored the relationships between resilience, safety attitude, and safety behavior. Studies have shown that resilience positively affects safety attitude and safe behavior. Safety attitude positively affects safety behaviors and plays a role as a partial mediator in the impact of resilience on safe behavior. The theoretical contribution is that the resilience of miners has a positive impact on safety behavior. Moreover, resilience can also act on safety behaviors through the partial intermediation of safety attitude. The practical contribution is that managers of coal mining companies can promote the resilience and safety attitude of coal miners to improve safety behaviors and prevent accidents.

## 1. Introduction

Due to the high-hazard and high-stress work environment, excessive labor intensity, family–work conflicts, and low social status, some underground coal miners commonly suffer from fear, low self-esteem, anxiety, and depression. Individual behavior is governed by intrinsic psychological factors. Then, the poor mental quality of coal miners is prone to unsafe behavior. Furthermore, unsafe behavior of coal miners is the leading cause of accidents [1]. Therefore, coal mine enterprises must pay close attention to the miners’ mental health, mainly underground workers. It is vital to study how to keep coal miners in an excellent psychological state and working safely in a high-hazard and high-stress environment. 

Resilience is an essential psychological resource that improves practitioners’ adaptability to high-hazard and high-stress environments. It can also significantly affect individual mental health, behavior, and organizational performance, and it helps people relieve stress and cope with daily challenges [2,3,4,5,6,7]. Moreover, resilience contains attitude, awareness, and the strategy to cope with damage [8]. Then, safety attitude reflects coal miners’ beliefs and emotions about safety procedures, practices, and policies [9,10,11,12], which affect whether coal miners follow safety regulations and operating procedures. To some extent, it determines the individual’s choice of safe behavior [13]. There are few studies related to coal miners’ resilience. Whether coal miners with high resilience typically exhibit correct safety attitude and proactive safety behaviors is urgent to study. 

Thus, this study takes a particular occupational group of coal miners as the research object who work in a specific underground environment for a long time and are prone to various stressors. It is unclear how resilience affects safety behavior and whether safety attitude can play a mediating role needs to be clarified. It is crucial to investigate the influence of coal miners’ resilience on safety behavior and the mediating effect of safety attitude from an empirical perspective.

## 2. Literature Review and Hypotheses Proposal

This section reviewed the research on resilience, safety behavior, and safety attitude. It proposed corresponding hypotheses that coal miners’ resilience significantly and positively affects safety behavior and that resilience can indirectly affect safety behavior by affecting safety attitude in the context of conservation of resources theory.

### 2.1. Resilience

Resilience has been studied for over 30 years and can be broadly defined in three ways. First, under the outcome definition, resilience is a phenomenon in which individuals adapt well or develop well in the face of serious threats [14]. Second, under the capability definition, resilience is the ability or trait of an individual to withstand high levels of disruptive change, recover from it, and adapt flexibly to the environment while exhibiting few undesirable behaviors [15,16]. Finally, under the process definition, resilience is the process of adapting well to recover from a harrowing experience when an individual faces adversity, trauma, tragedy, threat, or other life stressors [17]. From these different perspectives, resilience has two common definition elements. First, individuals are in trouble. Second, when faced with difficulties and pressures, individuals can adapt themselves or adapt using coping skills.

The main evaluation methods of resilience are self-reported questionnaires, projection, behavior, or symptom assessment. The self-reported questionnaire has been widely recognized. The Connor–Davidson resilience scale is a typical scale under the framework of trait theory or ability theory [18]. The scale is based on the result theory [19] and evaluates resilience from the perspective of protective factors [20]. 

Many researchers also studied the impact of resilience on other factors. Wu et al. found that resilience had preventive and protective effects on depression in left-behind children through longitudinal research [21]. Zhang et al. investigated the influence of resilience on the quality of life of breast cancer patients and the mediating role of social support. The results showed that resilience not only directly affected the quality of life of cancer patients but also indirectly affected their quality of life through social support [22]. In addition, Li et al. found that resilience is an essential protective factor for the elderly and plays a significant intermediary role between social support and senile depression in Singapore [23]. Hao et al. explored the mediating role of resilience in the relationship between neuroticism and the general well-being of the elderly. Moreover, psychological resilience reduced the negative effects of adverse mental states [24]. To sum up, resilience as a positive psychological resource can protect people from their negative states or emotions. However, resilience research mainly focuses on social and psychological fields, while there is less research on industry personnel, especially in the coal mining industry.

Based on the coal miners’ work characteristics, resilience was defined as the trait or ability of being able to recover when facing threats, adversities, or other life and work stressors [25]. Gao et al. revised and validated the resilience scale for coal miners [25], including two dimensions, i.e., tenacity and strength, that can effectively measure coal miners’ resilience in China.

### 2.2. Safety Behavior

Safety behavior was defined as individuals’ behaviors to protect their health and maintain workplace safety [26]. Safety behavior mainly includes safety participation and compliance. Safety participation refers to employees’ active involvement in maintaining workplace safety, for example, helping colleagues at work and improving workplace procedures. Safety compliance refers to complying with safety policies and regulations, following correct operating procedures, and using equipment and facilities correctly [27].

Factors of safety behavior include extrinsic and intrinsic factors. Internal factors include employees’ physical state, knowledge level, individual psychological characteristics [28,29,30], safety attitude [13], personality traits, etc. External factors include the work environment, job stress [31], safety climate [27,32,33], safety culture, colleagues’ behavior, and managers’ commitment to safety [34].

Safety behaviors are mainly measured by questionnaire surveys and observation. The definition and scale of safety behavior proposed by Griffin and Neal [27] were used.

### 2.3. Safety Attitude

Safety attitude is widely recognized as a part of safety culture [35]. The research on safety attitude mainly includes the definition, structure dimensions, scale, and factors that influence safety attitude.

Safety attitude was defined as employees’ perceptions of the importance of safety, emotions about the implementation of safety policies, and behavioral tendencies toward implementing safety regulations [10].

The structure dimensions of safety attitude can be broadly classified into two types. One encompasses the three structural dimensions of attitude under safety attitude [36]. The other constructs the structural dimensions of safety attitude based on its objects. Cox classified the objects of safety attitude into risk, people, safety software and concepts, safety hardware, and physical hazard [37]. 

Safety attitude scales and structure dimensions also vary across industries and regions. Williamson’s safety attitude questionnaire designed in the manufacturing industry is representative [38]. The Donald’s safety attitude scale developed in the chemical industry is widely used [36]. Wu et al. constructed a safety attitude scale for coal miners, including team safety climate, management safety commitment, job stress, and fatalism [10]. 

Safety attitude significantly affects whether employees follow safety regulations and comply with proper operating procedures. Moreover, safety attitude affects employees’ choice of safety behavior. The correct safety attitude of coal miners, especially the underground front-line workers, can improve safety behavior and prevent accidents. 

### 2.4. Hypotheses on the Relationship between Resilience, Safety Attitude, and Safety Behaviors

Based on the conservation of resources theory and the literature review, the following hypotheses were proposed.

Conservation of resources theory assumes that individuals always do their best to maintain, protect, or construct valuable resources. The loss of existing or potential resources threatens the individual [39]. Conservation of resources theory reveals the spiral effect of resources that people with more psychological resources have a greater capacity for tolerance and relief of stress [40]. Resilience, as a positive psychological resource and personality trait, can improve individuals’ adaptability to high-hazard and high-stress environments and help them relieve job stress and cope with difficulties and challenges in work and life [2]. 

When coal mining enterprises arrange additional work tasks for the miners, resilience will play a mitigating or inhibiting role, reduce the loss of individual coal miners’ mental resources, and recover depleted mental resources relatively quickly. 

Based on the definition of safe behavior and its influencing factors, behavior is influenced by psychological factors, and resilience can also affect an individual’s choice of safe behavior. The greater the resilience of coal miners, the more optimistic their safety behaviors. Coal miners with resilience (tenacity, strength, optimism, etc.) are capable of coping with job stress and work tasks. They will maintain a higher safety attitude and exhibit proactive safety behaviors. Therefore, the following hypotheses are proposed:

**Hypothesis 1** **(H1).** *Coal miners’ resilience significantly and positively affects their safety behavior.*

**Hypothesis 2** **(H2).** *Coal miners’ resilience significantly and positively affects their safety attitude.*

Based on previous research, safety attitude positively affects not only safety behavior [13,41]. Fugas found that the impact of organizational safety climate on active safety behaviors was mediated by safety attitude. Safety attitude affects safety participation through perceived behavioral control and indirectly influences safety compliance [42]. Differences in the safety attitudes of coal miners will influence their choice of safety behaviors when faced with emergencies. 

When encountering unexpected situations and facing adversity or negative emotions, coal miners with greater resilience can resolve underground emergencies, cope with job stress, and recover from negative psychological states more quickly. Resilience can partly act on unsafe behaviors through safety attitude. Thus, the following hypotheses were proposed.

**Hypothesis 3** **(H3).** *Safety attitude significantly and positively affects their safety behaviors.*

**Hypothesis 4** **(H4).** *Safety attitude plays a mediating role in the impact of coal miners’ resilience on their safety behaviors.*

## 3. Research Tools and Methods

This section introduces the selection of resilience, safety attitude, and safe behavior scales, the research objects and procedures of the questionnaire survey, and the statistical analysis methods used to verify assumptions.

### 3.1. Research Scale

This study used the resilience scale [25], safety attitude scale [10], and safety behavior scale [27], which had high reliability and validity. The study questionnaire includes the description of the purpose of the survey, the demographic variables, a work-type survey, and the above three scales. These scales adopt five-point Likert scales, that is, each question has five options, completely disagree, disagree, unclear, agree, and completely agree, corresponding to 1–5 points, respectively. The higher the score, the greater the resilience, safety behavior, and safety attitude. 

### 3.2. Samples and Procedures

The sample is consistent with the second test of the resilience scale [25]. The research objects were coal miners from Shaanxi, Shandong, and Inner Mongolia provinces. A total of 839 questionnaires were received. After deleting the questionnaires with missing values and invalid questionnaires with apparent tendencies, and then deleting the no-underground work experience according to the work-type, 639 valid questionnaires were obtained with a 76.2% response rate. The subjects were all male. The demographic information is shown in Table 1.

### 3.3. Data Analysis Method

SPSS Statistics 22.0 was used to analyze these scales. First, confirmatory factor analysis (CFA) was used to verify the validity and reliability of these scales. 

The KMO coefficient and Bartlett’s test of sphericity were used to measure each scale’s validity. The quality of the dimension structure of these scales was evaluated by the overall goodness of fit (GOF). Cronbach’s α was used to measure the internal consistency (i.e., reliability) [43] of scales and their dimensions.

To confirm the validity, the commonly used indicators of the GOF test were −χ^2^/df, root mean square error of approximation (RMSEA), Tucker-Lewis index (TLI), and comparative fit index (CFI) [44].

First correlation analysis and then hierarchical regression analysis were adopted to study the correlations and relationships between the three variables resilience, safety attitude, and safety behaviors among the group of coal miners. Finally, the mediating effect test was used to determine the role of safety attitude in the impact of resilience on safety behavior. 

The mediating role was tested in turn as follows. The regression coefficient *c* of resilience on safety behavior was obtained by hierarchical regression. After adding safety attitude as the mediating variable, the regression coefficient of resilience on safety attitude was *a*. The regression coefficient of the impact of safety attitude on safety behavior was *b*. Then, the regression coefficient of resilience on safety behavior was *c*′. 

If *c* is significant, the next test is whether *a* and *b* are significant. Then, if *a* and *b* are significant, the next test is whether *c*′ is significant. If *c*′ is significant, then safety attitude plays a partially mediating role; if *c*′ is not significant, safety attitude plays a fully mediating role. If *a*, *b* is insignificant, then the bootstrap method is used to directly test the coefficient product *ab* to improve the test power [45].

## 4. Results

This section used CFA to verify the validity and reliability of these scales. Then, hierarchical regression analysis was used to investigate the relationships between the resilience, safety attitude, and safety behavior of coal miners. Finally, hierarchical regression analysis and sequential tests were used to study the mediating effect of safety attitude in the impact of resilience on safety behavior.

### 4.1. Validity and Reliability of the Three Scales

The safety attitude scale [10], resilience scale [25], and safety behavior scale [27] for coal miners were verified, so reliability analysis and CFA were required. The analysis of the three scales is shown in Table 2.

The KMO values for the three scales were more than 0.8. At the same time, Bartlett’s sphericity test showed significance (*p* < 0.01), and factor analysis revealed that the correspondence between the structural dimensions of the three scales and their items was consistent with expectations. Meanwhile, the commonality of all the items of these scales was greater than 0.4. The cumulative explanation rate of the three scales was more than 50%, implying that the information content of the items could be effectively extracted. Moreover, the standard loading coefficients of each question item were all greater than 0.7, indicating the correspondence between the scale items and dimensions. All three scales were found to have high internal consistency. The results of the CFA are shown in Table 3.

In Table 3, the indicators of the resilience scale and safety behavior scale fit well with the standards. The indicators of the safety attitude scale had little difference from the acceptable value and matched the standards. The internal consistency coefficient, Cronbach’s alpha, was used to test the reliability of the three scales. The reliability results of the three scales and each dimension are shown in Table 4. 

The Cronbach’s alpha of the coal miner safety attitude scale was 0.657, indicating acceptable data. The reliability of the other scales and each dimension was greater than 0.7, which proves that the three scales have good reliability. 

The total number of items on the three scales was 29, and the sample was 639, with a variable-to-sample ratio of 1:22.03, which met the requirements for CFA [44].

In conclusion, the standardized loading coefficients of each item were greater than 0.7, showing strong correlations between the items and dimensions. Moreover, the results of CFA revealed a high degree of fit of the model indicators. The reliability test found that the reliability of the safety attitude scale was acceptable, and the reliability of the resilience scale and safety behavior scale was high. Therefore, these three scales can be used to measure the resilience, safety attitude, and safety behaviors of coal miners.

### 4.2. Correlation Analysis

Correlation analysis was used to study the correlation between age, education level, length of service, marital status, accident experience, resilience, safety attitude, and safety behavior. The Pearson correlation coefficient indicates the strength of the correlation. 

From Table 5, resilience had significant and positive correlations with safety attitude and safety behavior. Then, safety attitude significantly and positively correlated with safety behavior. In contrast, the length of service, marital status, and accident experience had insignificant correlations with resilience. Additionally, age negatively correlated with resilience. Education level significantly and positively correlated with resilience. 

From Table 6, the two dimensions of coal miners’ resilience significantly and positively correlated with the two dimensions of safety behavior.

Table 7 shows that management safety commitment and team safety climate significantly and positively correlated with safety compliance and safety participation. Job stress and fatalism had significant negative correlations with safety participation. Moreover, fatalism negatively correlated with safety compliance. Job stress did not correlate with safety compliance.

Table 8 shows significant and positive correlations between tenacity and management safety commitment; tenacity and management safety commitment; strength and management safety commitment; and strength and team safety climate. Then, tenacity and strength significantly and negatively correlated with fatalism and job stress. 

### 4.3. Regression Analysis of the Impact of Resilience on Safety Behavior

Hypothesis 1 was tested with hierarchical regression analysis. The coal miners’ resilience was the dependent variable, and age, education level, length of service, marital status, and accident experience were the control variables. Safety behavior was the independent variable in Model 2. The results of the hierarchical regression are shown in Table 9.

The result showed that age (regression coefficient *β* = 0.023, *t* = 0.720, *p* = 0.472 > 0.05), length of service (*β* = −0.026, *t* = −0.990, *p* = 0.323 > 0.05), and marital status (*β* = 0.039, *t* = 0.688, *p* = 0.492 > 0.05) did not show significance for safety behavior, indicating that these variables did not affect safety behavior. Education level (*β* = 0.059, *t* = 2.087, *p* = 0.037 < 0.05) significantly and positively affected safety behavior. Accident experience (*β* = −0.189, *t* = −2.841, *p* = 0.005 < 0.01) significantly and negatively affected safety behavior.

Model 2 showed a significant change in F (*p* < 0.05) after adding resilience as the dependent variable, indicating that resilience had explanatory significance in the model. In addition, R-squared increased from 0.024 to 0.325, indicating that resilience can have an explanatory strength of 30.1% on safety behavior. Resilience (*β* = 0.510, *t* = 16.775, *p* = 0.000 < 0.01) had a significant and positive effect on safety behavior. Hypothesis 1 was verified.

Then, the influence of two dimensions of resilience on two dimensions of safety behavior was explored, with safety compliance and safety participation as dependent variables; age, education level, length of service, marital status, and accident experience as control variables; and two dimensions of resilience, tenacity and strength, as independent variables in a hierarchical regression analysis. The results are shown in Table 10.

From Table 10, R-squared for model 3 was 0.008, which indicated that age, length of service, education, marital status, and accident experience could explain 0.8% of the variation in safety compliance. F testing (F = 0.993, *p* > 0.05) indicated that these variables did not affect safety compliance. 

After adding strength and tenacity as dependent variables, there was a significant change in F (*p* < 0.05), and R-squared increased from 0.008 to 0.236, indicating that strength and tenacity had significant explanatory strength of 22.8% for safety compliance. Tenacity (*β* = 0.126, *t* = 6.723, *p* = 0.000 < 0.01) and strength (*β* = 0.222, *t* = 4.150, *p* = 0.000 < 0.01) had a significant positive influence on safety compliance. 

R-squared for Model 5 was 0.032, reflecting that age, length of service, education level, marital status, and accident experience explained 3.2% of the variation in safety participation. The F test (F = 4.175, *p* < 0.05) indicated that at least one of age, length of service, education, marital status, or accident experience affected safety participation. Data showed that age, length of service (*β* = −0.031, *t* = −1.049, *p* = 0.295 > 0.05), and marital status (*β* = 0.074, *t* = 1.142, *p* = 0.254 > 0.05) did not affect safety participation. In contrast, education level (*β* = 0.081, *t* = 2.477, *p* = 0.014 < 0.05) significantly and positively affected safety participation, and accident experience (*β* = −0.246, *t* = −3.222, *p* = 0.001 < 0.01) significantly and negatively affected safety participation. 

After adding strength and tenacity as dependent variables, there was a significant change in F (*p* < 0.05) that indicated that it had explanatory significance in Model 6. R-squared increased from 0.032 to 0.323, indicating that tenacity and strength had explanatory strength of 29.1% for safety participation. The data showed that strength (*β* = 0.153, *t* = 3.621, *p* = 0.000 < 0.01) and tenacity (*β* = 0.424, *t* = 9.255, *p* = 0.000 < 0.01) positively influenced safety participation.

### 4.4. Regression Analysis of the Impact of Resilience on Safety Attitude

Here, resilience was the independent variable. Age, education level, length of service, marital status, and accident experience were control variables, and safety attitude was the dependent variable for hierarchical regression analysis. The results are shown in Table 11.

R-squared for model 1 was 0.001, which indicates that age, education level, length of service, marital status, and accident experience could explain 0.1% of the variation in safety attitude. Model 1 did not pass the F test (F = 0.148, *p* > 0.05): age, education level, length of service, marital status, and accident experience did not affect safety attitude. 

The significant change in F (*p* < 0.05) indicated that adding resilience as a dependent variable had explanatory significance in Model 2. In addition, R-squared increased from 0.001 to 0.029, indicating that resilience had explanatory strength of 2.8% for safety attitude. Resilience (*β* = 0.111, *t* = 4.268, *p* = 0.000 < 0.01) had a significant positive relationship with safety attitude. Therefore, hypothesis 2 was verified. 

Then, a hierarchical regression was conducted with tenacity and strength as independent variables; age, education level, length of service, marital status, and accident experience as control variables; and the four dimensions of safety attitude as dependent variables. The results are shown in Table 12. 

R-squared for model 3 was 0.021, which indicates that age, education level, length of service, marital status, and accident experience could explain 2.1% of the variation in management safety commitment. Model 3 passed the F test (F = 2.693, *p* < 0.05), which indicated that at least one of age, education level, length of service, marital status, or accident experience affects management safety commitment. Age (*β* = 0.063, *t* = 1.496, *p* = 0.135 > 0.05), length of service (*β* = −0.030, *t* = −0.880, *p* = 0.379 > 0.05), education level (*β* = 0.063, *t* = 1.690, *p* = 0.091 > 0.05), and marital status (*β* = 0.009, *t* = 0.125, *p* = 0.901 > 0.05) did not affect management safety commitment. Accident experience (*β* = −0.261) had a significant negative relationship with management safety commitment.

The significant change in F (*p* < 0.05) indicated that tenacity and strength as dependent variables had explanatory significance in Model 4. R-squared increased from 0.021 to 0.213, indicating that tenacity and strength can explain 19.2% of the strength of management safety commitment. Tenacity (*β* = 0.365, *t* = 6.493, *p* = 0.000 < 0.01) and strength (*β* = 0.170, *t* = 3.283, *p* = 0.001 < 0.01) significantly and positively affected management safety commitment. R-squared for model 5 was 0.017, indicating that age, education level, length of service, marital status, and accident experience explained 1.7% of the variation in team safety climate. Model 5 did not pass the F test (F = 2.179, *p* > 0.05): age, education level, length of service, marital status, and accident experience did not affect team safety climate. The significant change in F (*p* < 0.05) indicated that adding tenacity and strength as dependent variables had explanatory significance in Model 6. R-squared increased from 0.017 to 0.191, indicating that tenacity and strength could explain 17.4% of the strength of the team safety climate. Tenacity (*β* = 0.283, *t* = 5.945, *p* = 0.000 < 0.01) and strength (*β* = 0.143, *t* = 3.252, *p* = 0.001 < 0.01) positively and significantly affected team safety climate. 

Then, tenacity and strength were used as independent variables; age, education level, length of service, marital status, and accident experience were control variables; and fatalism and job stress were dependent variables. The result of the hierarchical regression analysis is shown in Table 13. 

The dependent variable in Model 7 and Model 8 was fatalism. R-squared for Model 7 was 0.013, indicating that age, education level, length of service, marital status, and accident experience explained 1.3% of the variation in fatalism. Model 7 did not pass the F test (F = 1.698, *p* > 0.05): age, education level, length of service, marital status, and accident experience did not affect fatalism. 

The significant change in F (*p* < 0.05) indicated that adding tenacity and strength as independent variables had explanatory significance in Model 8. Moreover, R-squared increased from 0.013 to 0.045, reflecting that tenacity and strength had explanatory strength of 3.2% for fatalism. Tenacity (*β* = −0.241, *t* = −3.150, *p* = 0.002 < 0.01) had a significant negative relationship with fatalism, and strength (*β* = −0.018) did not affect fatalism. 

The dependent variable in Model 9 and Model 10 was job stress, and R-squared for Model 9 was 0.021, indicating that age, education level, length of service, marital status, and accident experience could explain 2.1% of the variation in job stress. Model 9 passed the F test (F = 2.676, *p* < 0.05): at least one of age, education level, length of service, marital status, or accident experience affected job stress. Age (*β* = 0.012, *t* = 0.287, *p* = 0.774 > 0.05), length of service (*β* = 0.014, *t* = 0.424, *p* = 0.671 > 0.05), education level (*β* = 0.001, *t* = 0.026, *p* = 0.979 > 0.05), and marital status (*β* = −0.067, *t* = −0.934, *p* = 0.351 > 0.05) did not affect job stress. Accident experience (*β* = 0.263, *t* = 3.129, *p* = 0.002 < 0.01) had a significant positive impact on job stress. 

The significant change in F (*p* < 0.05) indicated that adding tenacity and strength as dependent variables had explanatory significance in Model 10. R-squared increased from 0.021 to 0.054, indicating that tenacity and strength had explanatory strength of 3.4% for job stress. Specifically, tenacity (*β* = −0.203, *t* = −3.418, *p* = 0.001 < 0.01) had a significant negative relationship with job stress, and strength (*β* = −0.004) did not affect job stress.

### 4.5. Regression Analysis of the Impact of Safety Attitude on Safety Behavior

Hierarchical regression analysis was conducted with safety attitude as the independent variable; age, education level, length of service, marital status, and accident experience as control variables; and safety behavior as the dependent variable. The results are shown in Table 14. 

R-squared for model 1 was 0.024, which indicated that age, education level, length of service, marital status, and accident experience explained 2.4% of the variation in safety behavior. Model 1 passed the F test (F = 3.090, *p* < 0.05), reflecting that at least one of age, education level, length of service, marital status, or accident experience affected safety behavior. Age (*β* = 0.023, *t* = 0.720, *p* = 0.472 > 0.05), length of service (*β* = −0.026, *t* = −0.990, *p* = 0.323 > 0.05), and marital status (*β* = 0.039, *t* = 0.688, *p* = 0.492 > 0.05) did not have an impact on safety behavior. Education level (*β* = 0.059, *t* = 2.087, *p* = 0.037 < 0.05) significantly and positively affected safety behavior, and accident experience (*β* = −0.189, *t* = −2.841, *p* = 0.005 < 0.01) significantly and negatively affected safety behavior. 

The significant change in F (*p* < 0.05) indicated that adding safety attitude as the dependent variable had explanatory significance in Model 2. R-squared increased from 0.024 to 0.078, indicating that safety attitude had explanatory strength of 5.4% for safety behavior. Safety attitude (*β* = 0.326, *t* = 6.111, *p* = 0.000 < 0.01) significantly and positively affected safety behavior. Therefore, hypothesis 3 was verified. 

Then, the relationship between the dimensions of safety attitude and safety behavior was investigated. The four dimensions of safety attitude were the independent variables; age, education level, length of service, marital status, and accident experience were the control variables; and safety participation and safety compliance were the dependent variables for hierarchical regression. The results are shown in Table 15.

R-squared for model 3 was 0.008, which indicated that age, education level, length of service, marital status, and accident experience could explain 0.8% of the variation in safety compliance. Model 3 did not pass the F test (F = 0.993, *p* > 0.05): age, education level, length of service, marital status, and accident experience did not affect safety compliance. 

The significant change in F (*p* < 0.05) indicated that adding management safety commitment, fatalism, team safety climate, and job stress as independent variables had explanatory significance in Model 4. R-squared increased from 0.008 to 0.415, indicating that management safety commitment, fatalism, team safety climate, and job stress had explanatory strength of 40.7% for safety compliance. Management safety commitment (*β* = 0.153, *t* = 6.655, *p* = 0.000 < 0.01), team safety climate (*β* = 0.333, *t* = 12.395, *p* = 0.000 < 0.01), and job stress (*β* = 0.061, *t* = 2.932, *p* = 0.003 < 0.01) significantly and positively affected safety compliance. In contrast, fatalism (*β* = −0.012) did not affect safety compliance. 

R-squared for Model 5 was 0.032, indicating that age, education level, length of service, marital status, and accident experience explained 3.2% of the variation in safety participation. The F test (F = 4.175, *p* < 0.05) indicated that at least one of age, education level, length of service, marital status, or accident experience affected safety participation. Age (*β* = 0.026, *t* = 0.710, *p* = 0.478 > 0.05), length of service (*β* = −0.031, *t* = −1.049, *p* = 0.295 > 0.05), and marital status (*β* = 0.074, *t* = 1.142, *p* = 0.254 > 0.05) did not affect safety participation. In contrast, education level (*β* = 0.081, *t* = 2.477, *p* = 0.014 < 0.05) significantly and positively affected safety participation, and accident experience (*β* = −0.246, *t* = −3.222, *p* = 0.001 < 0.01) significantly and negatively affected safety participation. 

The significant change in F (*p* < 0.05) indicated that adding management safety commitment, fatalism, team safety climate, and job stress as dependent variables had explanatory significance in Model 6. Meanwhile, R-squared increased from 0.032 to 0.361, indicating that management safety commitment, fatalism, team safety climate, and job stress had explanatory strength of 32.9% on safety participation. Management safety commitment (*β* = 0.170, *t* = 4.819, *p* = 0.000 < 0.01) and team safety climate (*β* = 0.451, *t* = 10.929, *p* = 0.000 < 0.01) significantly and positively affected safety participation. In contrast, fatalism (*β* = −0.040) and job stress (*β* = −0.046) did not affect safety participation.

### 4.6. The Mediating Role of Safety Attitude in the Impact of Resilience on Safety Behavior

This section studied the mediating role of safety attitude whereby resilience affects safety behavior. Age, education level, length of service, marital status, and accident experience as control variables were standardized. Model 1 used resilience as the independent variable and safety attitude as the dependent variable. Model 2 used resilience as the independent variable and safety behavior as the dependent variable. Model 3 used resilience and safety attitude as independent variables and safety behavior as the dependent variable. Then, the mediating role of safety attitude was explored using hierarchical regression analysis and a mediating role test. The results are presented in Table 16.

It was first measured whether the resilience of coal miners was significant for safety behavior, and the results showed that resilience significantly and positively affected safety behavior (*β* = 0.556, *t* = 16.775, *p* < 0.01). Then, the impact of resilience on safety attitude and safety attitude on safety behaviors was measured. Data analysis showed a significant and positive relationship between resilience and safety attitude (*β* = 0.170, *t* = 4.628, *p* < 0.01) and a significant and positive relationship between safety attitude and safety behaviors (*β* = 0.146, *t* = 4.458, *p* < 0.01). 

Finally, the impact of resilience on safety behavior was measured after adding safety attitude as a mediating variable. The data showed that resilience significantly and positively affected safety behavior (*β* = 0.532, *t* = 16.037, *p* < 0.01) after the inclusion of safety attitude. To sum up, safety attitude plays a partially mediating role in the impact of resilience on the safety behavior of coal miners. Therefore, hypothesis 4 was verified. 

Then, age, education level, length of service, marital status, and accident experience were used as control variables. Model 4 took resilience as the independent variable and safety compliance as the dependent variable. Model 5 used resilience and safety attitude as independent variables and safety compliance as the dependent variable. Hierarchical regression was used to study the mediating role of safety attitude. The result is shown in Table 17.

The table shows that resilience significantly and positively affected safety compliance (*β* = 0.484, *t* = 13.716, *p* < 0.01). Next, resilience significantly and positively affected safety attitude (*β* = 0.170, *t* = 4.628, *p* < 0.01), and safety attitude was significantly and positively related to safety compliance (*β* = 0.321, *t* = 9.753, *p* < 0.01). Finally, after adding safety attitude as the mediating variable, resilience was significantly and positively related to safety compliance (*β* = 0.430, *t* = 12.862, *p* < 0.01). In summary, safety attitude plays a partially mediating role in the relationship between resilience and safety compliance. 

Finally, the mediating role of safety attitude in the impact of resilience on safety participation was investigated. Model 6 used resilience as the independent variable and safety participation as the dependent variable. In Model 7, safety attitude and resilience were the independent variables, and safety participation was the independent variable. Based on the above conditions, hierarchical regression analysis was conducted on the mediating role of safety attitude in the impact of resilience on safety behaviors. The results are shown in Table 18.

The table indicated that resilience was significantly and positively related to safety participation (*β* = 0.542, *t* = 16.243, *p* < 0.01). Resilience was also significantly and positively related to safety attitude (*β* = 0.170, *t* = 4.628, *p* < 0.01), and safety attitude was significantly and positively related to safety participation (*β* = 0.158, *t* = 4.826, *p* < 0.01). Finally, after adding safety attitude as the mediating variable, resilience significantly and positively affected safety participation (*β* = 0.515, *t* = 15.486, *p* < 0.01). In summary, safety attitude plays a partially mediating role in the impact of coal miners’ resilience on safety participation.

## 5. Discussion

The relationships between resilience, safety attitude, and safety behaviors of coal miners were analyzed; the impacts of demographic variables on resilience, safety attitude, and safety behaviors were analyzed; and some measures were proposed to promote safety for coal mining enterprises.

### 5.1. Relationships between Resilience, Safety Attitude, and Safety Behaviors of Coal Miners

Based on correlation analysis and hierarchical regression analysis, it can be concluded that coal miners’ resilience positively affects safety behavior. 

The impact of two dimensions of resilience on safety participation and safety compliance was also investigated. The regression coefficients for tenacity and strength on safety compliance were 0.222 and 0.126, and *p* for both was less than 0.01. It indicated that tenacity and strength positively affected safety compliance. Moreover, the regression coefficients for tenacity and strength on safety participation were 0.424 and 0.153, and *p* for both was less than 0.01. This showed that tenacity and strength positively influenced safety participation. Thus, both tenacity and strength significantly and positively affected both safety participation and safety compliance. The impact of tenacity on safety participation and safety compliance was greater than that of strength. When encountering unexpected situations and suffering job stress, coal miners with tenacity can relieve the stress and spontaneously comply with safety procedures and regulations. Coal miners with tenacity serve as role models and motivators for their coworkers. When experiencing accidents, hardship, or adversity, coal miners with strength can recover from the trauma or negative emotions and deal with problems at work and therefore also exhibit safety participation and safety compliance behaviors. 

Resilience is significantly and positively related to safety attitude (*β* = 0.111, *p* < 0.01). The impact of the two dimensions of resilience on the four dimensions of safety attitude was also investigated, and the results showed that the regression coefficients of tenacity (*β* = 0.365, *p* < 0.01) and strength (*β* = 0.170, *p* < 0.01) positively and significantly affected management safety commitment. 

Combined with the correlation analysis, tenacity (*β* = 0.283, *p* < 0.01) and strength (*β* = 0.143, *p* < 0.01) had significant and positive relationships with team safety climate. Moreover, tenacity significantly and negatively affected fatalism (*β* = −0.241, *p* < 0.01) and job stress (*β* = −0.203, *p* < 0.01). Because strength had a negative correlation but an insignificant relationship with job stress and fatalism, strength did not affect fatalism and job stress.

Similar to the findings of Shin and Zhang et al. [46,47], safety attitude (*β* = 0.326, *p* < 0.01) significantly and positively affected safety behaviors. The regression coefficients of team safety climate, management safety commitment, and job stress with safety compliance were 0.333, 0.153, and 0.061, respectively, and positively influenced safety compliance at significance of 0.01. Additionally, fatalism did not affect safety compliance (*β* = −0.012), and because job stress was not correlated with safety compliance, job stress did not affect safety compliance. 

Fatalism had a negative correlation but no relationship with safety compliance. Management safety commitment (*β* = 0.170, *p* < 0.01) and team safety climate (*β* = 0.451, *p* < 0.01) positively affected safety participation. Fatalism and job stress had negative correlations but no relationships with safety participation. In addition, management safety commitment significantly affected safety participation and safety compliance, which was consistent with Rundmo and Zhang’s studies [48,49]. Li et al. found an insignificant relationship between management safety commitment and safety participation [13], which is slightly different from the findings of this study. Due to managers’ commitment to safety, coal miners are willing to participate spontaneously in maintaining workplace safety. It may be why management safety commitment influenced coal miners’ safety participation. If managers encourage coal miners to discuss workplace safety issues and provide protective equipment, coal miners’ motivation for safety participation will also increase.

Resilience significantly and positively affected safety behavior (*p* < 0.01) and safety attitude (*p* < 0.01), safety attitude in turn significantly and positively affected safety behaviors (*p* < 0.01). After adding safety attitude as a mediating variable, resilience showed a significant and positive influence on safety behavior (*p* < 0.01). Thus, resilience can influence safety behavior by affecting safety attitude, and safety attitude plays a partially mediating role in the impact of coal miners’ resilience on safety behaviors. Additionally, safety attitude plays a partial mediating role in the effects of resilience on safety compliance and safety participation.

This study has practical implications for managers in coal mining enterprises. First, managers should focus on the resilience of coal miners and promptly reduce job stress. By setting up a mental health counseling room, coal miners can timely relieve stress and possible psychological problems at work. Especially for those coal miners who have experienced accidents, timely psychological guidance can help them recover from bad psychological states or negative emotions. 

Next, managers’ positive attitudes and commitment to safety will improve coal miners’ safety attitude and promote their safety behaviors. Managers are supposed to communicate frequently with their employees to understand coal miners’ needs and provide appropriate personal protection. 

In addition, praising and rewarding coal miners with proactive safety behaviors will motivate them to become spontaneously involved in maintaining workplace safety. Coal miners are likely to take unsafe actions when they believe improper actions will get the job done quickly and will not result in an accident. Coal miners who perform risky operations without admonition may induce other coal miners to violate safety procedures, rules, and regulations. Therefore, it is vital to promptly supervise coal miners who violate protocols. Moreover, targeted safety training and safety education are necessary. Through safety training and education, coal miners can recognize that accidents can be prevented and that coworker violations can jeopardize workplace safety. Coal miners can stop and discourage their coworkers who are operating in violation of the safety rules. 

Poor working conditions such as humidity and sweltering heat underground increase coal miners’ stress. Therefore, improving the working environment can reduce job stress and strengthen the resilience of coal miners. 

In summary, coal miners’ resilience significantly and positively affects safety behavior, and resilience also positively acts on safety behavior by affecting safety attitude. The greater the coal miners’ resilience, the better the safety attitude and the safety behavior. 

### 5.2. Impacts of Demographic Variables on Coal Miners’ Resilience, Safety Attitude, and Safety Behaviors

This study investigated the impact of demographic variables on the resilience, safety attitude, and safety behaviors of coal miners. Age had a negative correlation but no relationship with resilience and fatalism. Age and length of service did not affect resilience, safety attitude, and safety behaviors. Siu found that the older the age, the better the safety attitude [26]. It is likely that the safety climate and safety culture of different coal mining companies largely weaken the impact of age on coal miners’ safety attitude. Older coal miners are familiar with workplace procedures and hazards; thus, they may have a slightly higher safety attitude and safety behaviors than younger coal miners. The older coal miners had lower levels of fatalism than the younger ones, possibly because after safety education and training, the older coal miners believed that accidents could be prevented by safety practices. If older coal miners cannot recover from the negative psychological state caused by accident experience, their resilience may remain low. In conclusion, length of service and age have insignificant impacts on resilience, safety behavior, and safety attitude.

Education level showed a significant and positive effect on resilience and safety behaviors but did not affect safety attitude. This was similar to the findings of Li et al. that education level was not correlated with safety attitude [13]. The higher the education level, the higher the resilience of coal miners and the higher the safety behavior. Additionally, education level positively influenced safety participation and strength, and education level had a positive correlation but no relationship with tenacity. One possible reason is how coal miners view adversity or negative emotions related to education level. Coal miners with high education levels may view problems at work dialectically, face them with a healthy and optimistic mind, and choose reasonable methods to solve them. Moreover, coal miners can recover from negative emotions and gain experience from difficulties. Highly educated coal miners are likely to proactively participate in maintaining workplace safety and therefore behave at a higher level of safety behavior.

Marital status did not correlate with the resilience of coal miners, but it did have significant positive effects on resilience and strength. Marital status also did not affect safety attitude or safety behaviors. The impact of marital status on safety behavior was less significant under the overall safety climate. Married coal miners may be supported by their families and recover easily from negative emotions. They appear more confident and tenacious when dealing with stress or poor mental states, showing higher levels of strength.

Accident experience showed a significant and negative impact on safety behavior and no significant effect on resilience or safety attitude. Accident experience negatively affected safety participation and management safety commitment and positively affected job stress. Accident experience showed a significant and negative correlation with team safety climate. Coal miners with accident experience show less safety participation and have less trust in management safety commitment than coal miners without accident experience. Furthermore, coal miners with accident experience have greater job stress. One possible reason is that coal miners who have experienced accidents subconsciously worry about hazards, which increases their job stress. Then, accident experience could affect the team safety climate to some extent. Therefore, managers should pay attention to coal miners with accident experiences and provide them with appropriate safety education and psychological guidance. It can help coal miners recover from poor psychological states or negative emotions, which can be very helpful in improving safety behavior. Furthermore, managers can reasonably arrange positions and work tasks based on education level and accident experience.

In conclusion, the effects of demographic variables on resilience, safety attitude, and safety behavior may change with regions, industries, enterprises, and working environment, which needs further study. 

### 5.3. The Resilience, Safety Attitude, and Safety Behavior of Coal Miners

This study investigated the resilience, safety behaviors, and safety attitude of coal miners. Based on their scale scores, the coal miners in this study have good levels of resilience and safety attitude and high levels of safety behaviors. However, some areas still need to improve.

The two dimensions of the resilience of coal miners, strength and optimism, are at the upper middle level. Still, when faced with dangerous circumstances or accidents, coal miners might find it challenging to respond properly. Safety training about handling underground emergencies is necessary. One correct handling of an underground emergency may well prevent an accident. 

A low score on the resilience scale (“I know where to go for help) indicated that some coal miners did not know where to go for help with work-related stress, negative emotions, or trauma after an accident. In addition to support from coal miners’ colleagues, friends, and family, miners should receive support from their managers.

In the dimensions of coal miners’ safety attitude, management safety commitment is in the middle to upper level. These coal miners reported a lack of good communication between managers and employees. The opinions of coal miners are not well taken into account. Therefore, managers should appropriately improve communication with coal miners. Furthermore, coal miners with good safety behavior and high motivation can be rewarded with bonus incentives and promotion opportunities. 

The team safety climate is in the middle to upper range. It is vital that coal miners tend to resort to violations and infractions when they believe that disobeying the rules will save time and energy to get the job done quickly. Coal miners who commit a violation once are likely to do so again if they are not chastised by coworkers or disciplined by line managers. If other workers see that violators are not punished, they are likely to also commit violations, increasing the risk of accidents. Therefore, during safety education and safety training, educators should emphasize the dangers of noncompliance and encourage coal miners to monitor each other, emphasizing that the cost and consequences of noncompliance can be unbearable. 

Coal miners hold a moderate level of fatalism. Therefore, the theory of accident causation and prevention can be explained in detail such that coal miners know that safety practices can prevent accidents. At the same time, coal miners believe that their actions will prevent accidents more than those taken by managers. Thus, managers can improve coal miners’ safety attitude through encouraging them to participate in safety goals setting and safety activities. 

Additionally, coal miners’ job stress is at a medium level. Job stress mainly comes from poor working conditions and heavy workloads. Therefore, managers can reduce the job stress of coal miners by improving the working environment and arranging work tasks reasonably.

Finally, coal miners’ safety behavior levels are high, and safety compliance levels are higher than safety participation levels. Therefore, managers can provide incentives or rewards to increase coal miners’ motivation to maintain workplace safety, improve their safety compliance, and thereby improve their safety behavior overall.

### 5.4. Limitations and Future Research 

In particular, resilience is a dynamic ability. After experiencing an accident, longitudinal studies of changes in individual coal miners’ resilience are necessary. Cross-sectional studies cannot accurately explain the causal mechanisms between resilience, safety behaviors, and safety attitude, in particular, whether coal miners’ resilience, safety behaviors, and safety attitude would change after experiencing an accident. Resilience is a dynamic trait. Although this study found no significant difference in the impact of accident experience on resilience, longitudinal studies need to further confirm how accident experience will affect changes in resilience, safety attitude, and safety behaviors.

Self-report questionnaires can lead to social desirability bias. However, because of the assurance of anonymity and confidentiality on the questionnaires, social desirability bias was relatively low. Separately, respondents rated items on five-point Likert scales. Previous studies have found that Chinese subjects tend to select intermediate values when completing questionnaires [50], but this trend was not evident in this study. To avoid this ambiguity, all variables, except demographic variables, could be measured using 6-point or 10-point scales. This would make the data more distinguishable and facilitate subsequent analysis.

Finally, the coal miners’ work types were collected, and the miners with underground work experience were selected for exploring their resilience, safety attitude, and behavior. The influences of different work types on resilience, safety attitude and behavior need further study.

## 6. Conclusions

This study used the revised resilience scale, the safety attitude scale, and the mature safety behavior scale to study the impact of coal miners’ resilience on safety behavior and the mediating role of safety attitude. The main findings of the study were as follows.

(1) The resilience scale, safety attitude scale, and safety behavior scale for coal miners have good validity and reliability.
(2) Coal miners’ resilience significantly and positively influenced safety behavior. Additionally, the two dimensions of resilience significantly and positively influenced the two dimensions of safety behavior, and tenacity had a impact on safety participation and safety compliance than strength.
(3) Resilience positively affected safety attitude. Moreover, tenacity and strength significantly affected team safety climate and management safety commitment. Tenacity negatively affected fatalism and job stress.
(4) Safety attitude positively influenced safety behavior: there were significant relationships between each dimension of safety attitude and safety behavior. Team safety climate and management safety commitment positively affected safety participation and safety compliance. Job stress did not correlate with safety compliance. Job stress had a negative correlation but no relationship with safety participation. Fatalism had negative correlations but no relationships with safety compliance or safety participation.
(5) Safety attitude played a partial mediating role in the impact of resilience on safe behavior. Furthermore, safety attitude played a partial mediating role in the effects of resilience on two kinds of safe behavior.
(6) The effects of demographic variables on resilience, safety attitude, and safety behaviors of coal miners were investigated. Education level positively affected resilience and safety behavior, accident experience negatively affected safety behavior and safety participation, and management safety commitment positively affected job stress.

The theoretical contribution is the finding that coal miners’ resilience can influence safety behaviors directly and indirectly through safety attitude. The practical contribution emphasizes that managers should pay attention to coal miners with accident experience. It is recommended that managers provide miners with psychological guidance that can effectively relieve job stress and improve their resilience to recover from negative emotions or distress. Proper safety training and education as well as prompt communication are vital. With these supports in place, coal miners with high resilience will maintain good safety attitudes, strengthen participation in improving workplace safety, and ultimately prevent accidents.

## Figures and Tables

**Table 1:** Demographic information of coal miners.

Items	Sample Characteristics	Number	Percentage (%)
Age	25 years old and below	10	1.56
	26–30 years old	141	22.07
	31–35 years old	212	33.18
	36–40 years old	89	13.93
	41–45 years old	61	9.55
	46–50 years old	75	11.74
	51 years old and above	51	7.88
Length of service	5 years and below	121	18.94
	6–10 years	170	26.60
	11–15 years	136	21.28
	16–20 years	52	8.14
	21–25 years	43	6.73
	26–30 years	55	8.61
	31 years and above	62	9.70
Educational level	Junior Middle school education and below	42	6.57
	Higher school education	218	34.12
	Junior college degree	249	38.97
	Bachelor’s degree	122	19.09
	Master’s degree and above	8	1.25
Marital Status	Unmarried	70	10.95
	Married	556	87.01
	Divorced	13	2.03
Accident experience	No	546	85.45
	yes	93	14.55

**Table 2:** KMO coefficient, Bartlett’s test, and factor analysis of the three scales.

Scale (Number of Items)	KMO Value	Bartlett’s Spherical Test Significant Level	Dimension (Number of Items)	Explanation Rates	Cumulative Explanation Rate
Standard Value	>0.8				>50%
Resilience Scale (6)	0.887	*p* < 0.01	Tenacity (4)	40.889%	71.605%
			Strength (2)	30.716%	
Safety Attitude Scale (17)	0.837	*p* < 0.01	Management Safety Commitment (5)	18.697%	61.153%
			Fatalism (4)	15.407%	
			Team Safety Climate (4)	14.732%	
			Job Stress (4)	12.318%	
Safety Behavior Scale (6)	0.882	*p* < 0.01	Safety Compliance (3)	38.666%	76.716%
			Safety Participation (3)	38.050%	

**Table 3:** The result of the CFA.

Fit Index	X^2^(df)	X^2^/df	CFI	TLI	RMSEA
Standard Value [44]	<3	<3	≥0.95	≥0.95	<0.08
Resilience Scale	16.152(8) ***	2.019	0.995	0.991	0.040
Safety Attitude Scale	391.714(113) ***	3.466	0.929	0.915	0.062
Safety Behavior Scale	20.525(8) **	2.567	0.994	0.989	0.050

Note: *** indicate *p* < 0.001; ** indicate *p* < 0.01.

**Table 4:** Results of the reliability test of the three scales.

Scale (Number of Items)	Dimension (Number of Items)	Cronbach’s Alpha	
Resilience Scale (6)	Tenacity (4)	0.833	0.872
	Strength (2)	0.759	
Safety Attitude Scale (17)	Management Safety Commitment (5)	0.840	0.657
	Fatalism (4)	0.787	
	Team Safety Climate (4)	0.702	
	Job Stress (4)	0.811	
Safety Behavior Scale (6)	Safety Compliance (3)	0.856	0.892
	Safety Participation (3)	0.837	

**Table 5:** Correlation coefficients of the variables.

Variables	Age	Length of Service	Education Level	Marital Status	Accident Experience	Resilience	Safety Attitude	Safety Behavior
**Age**	1							
**Length of Service**	0.881 **	1						
**Academic Degree**	−0.359 **	−0.259 **	1					
**Marital Status**	0.296 **	0.250 **	−0.153 **	1				
**Accident Experience**	0.117 **	0.146 **	−0.056	0.056	1			
**Resilience**	−0.080 *	−0.063	0.120 **	0.070	−0.049	1		
**Safety Attitude**	0.006	0.001	−0.010	−0.024	−0.016	0.161 **	1	
**Safety Behavior**	−0.047	−0.060	0.090 *	0.006	−0.121 **	0.559 **	0.234 **	1

Note: * indicate *p* < 0.05. ** indicate *p* < 0.01.

**Table 6:** Correlation coefficients of coal miners’ resilience and safety behavior.

Dimensions	Tenacity	Strength
**Safety Compliance**	0.457 **	0.416 **
**Safety Participation**	0.539 **	0.464 **

Note: ** indicate *p* < 0.01.

**Table 7:** Correlation coefficients between safety attitude and safety behaviors.

Dimensions	Management Safety Commitment	Fatalism	Team Safety Climate	Job Stress
**Safety Compliance**	0.498 **	−0.083 *	0.607 **	0.010
**Safety Participation**	0.463 **	−0.152 **	0.549 **	−0.141 **

Note: * indicate *p* < 0.05. ** indicate *p* < 0.01.

**Table 8:** Correlation coefficients between resilience and safety behavior.

Dimensions	Management Safety Commitment	Fatalism	Team Safety Climate	Job Stress
**Tenacity**	0.426 **	−0.178 **	0.397 **	−0.191 **
**Strength**	0.377 **	−0.129 **	0.350 **	−0.141 **

Note: ** indicate *p* < 0.01.

**Table 9:** Regression analysis of resilience on safety behavior (N = 639).

Variable	Safety Behavior	
	Model 1	Model 2
**Age**	0.023 (0.720)	0.040 (1.501)
**Length of Service**	−0.026 (−0.990)	−0.029 (−1.328)
**Education Level**	0.059 (2.087 *)	0.019 (0.783)
**Marital Status**	0.039 (0.688)	−0.045 (−0.951)
**Accident Experience**	−0.189 (−2.841 **)	−0.150 (−2.712 **)
**Resilience**		0.510 (16.775 **)
**R^2^**	0.024	0.325
**Value of F**	F (5,633) = 3.090, *p* = 0.009	F (6,632) = 50.617, *p* = 0.000
**△R^2^**	0.024	0.301
**Value of △F**	F (5,633) = 3.090, *p* = 0.009	F (1,632) = 281.408, *p* = 0.000

Note: * indicate *p* < 0.05, ** indicate *p* < 0.01, T value is in parentheses.

**Table 10:** Regression analysis of the two dimensions of resilience on the two dimensions of safety behavior (N = 639).

Variable	Safety Compliance		Safety Participation	
	Model 3	Model 4	Model 5	Model 6
**Age**	0.016 (0.628)	0.027 (1.209)	0.026 (0.710)	0.043 (1.393)
**Length of Service**	−0.015 (−0.709)	−0.016 (−0.910)	−0.031 (−1.049)	−0.034 (−1.357)
**Education Level**	0.022 (0.966)	−0.005 (−0.268)	0.081 (2.477 *)	0.038 (1.387)
**Marital Status**	−0.013 (−0.292)	−0.067 (−1.699)	0.074 (1.142)	−0.008 (−0.148)
**Accident Experience**	−0.089 (−1.700)	−0.064 (−1.388)	−0.246 (−3.222 **)	−0.208 (−3.243 **)
**Strength**		0.126 (4.150 **)		0.153 (3.621 **)
**Tenacity**		0.222 (6.723 **)		0.424 (9.255 **)
**R^2^**	0.008	0.236	0.032	0.323
**F**	F (5,633) = 0.993, *p* = 0.421	F (7,631) = 27.871, *p* = 0.000	F (5,633) = 4.175, *p* = 0.001	F (7,631) = 43.007, *p* = 0.000
**△R^2^**	0.008	0.228	0.032	0.291
**△F**	F (5,633) = 0.993, *p* = 0.421	F (2,631) = 94.334, *p* = 0.000	F (5,633) = 4.175, *p* = 0.001	F (2,631) = 135.646, *p* = 0.000

Note: * indicate *p* < 0.05. ** indicate *p* < 0.01.

**Table 11:** Regression analysis of resilience on safety attitude (N = 639).

Variable	Safety Attitude	
	Model 1	Model 2
**Age**	0.007 (0.287)	0.010 (0.452)
**Length of Service**	−0.003 (−0.179)	−0.004 (−0.217)
**Education Level**	−0.005 (−0.243)	−0.014 (−0.681)
**Marital Status**	−0.028 (−0.679)	−0.046 (−1.135)
**Accident Experience**	−0.019 (−0.390)	−0.010 (−0.217)
**Resilience**		0.111 (4.268 **)
**R^2^**	0.001	0.029
**F**	F (5,633) = 0.148, *p* = 0.981	F (6,632) = 3.163, *p* = 0.005
**△R^2^**	0.001	0.028
**△F**	F (5,633) = 0.148, *p* = 0.981	F (1,632) = 18.219, *p* = 0.000

Note: ** indicate *p* < 0.01.

**Table 12:** Regression analysis of two dimensions of resilience on management safety commitment and team safety climate (N = 639).

Variable	Management Safety Commitment		Team Safety Climate	
	Model 3	Model 4	Model 5	Model 6
**Age**	0.063 (1.496)	0.080 (2.095 *)	0.041 (1.163)	0.055 (1.689)
**Length of Service**	−0.030 (−0.880)	−0.033 (−1.067)	−0.032 (−1.124)	−0.034 (−1.320)
**Education Level**	0.063 (1.690)	0.023 (0.668)	−0.018 (−0.574)	−0.051 (−1.767)
**Marital Status**	0.009 (0.125)	−0.070 (−1.051)	−0.034 (−0.545)	−0.098 (−1.728)
**Accident Experience**	−0.261 (−2.990 **)	−0.224 (−2.851 **)	−0.209 (−2.851 **)	−0.179 (−2.682 **)
**Strength**		0.170 (3.283 **)		0.143 (3.252 **)
**Tenacity**		0.365 (6.493 **)		0.283 (5.945 **)
**R^2^**	0.021	0.213	0.017	0.191
**F**	F (5,633) = 2.693, *p* = 0.020	F (7,631) = 24.384, *p* = 0.000	F (5,633) = 2.179, *p* = 0.055	F (7,631) = 21.276, *p* = 0.000
**△R^2^**	0.021	0.192	0.017	0.174
**△F**	F (5,633) = 2.693, *p* = 0.020	F (2,631) = 76.996, *p* = 0.000	F (5,633) = 2.179, *p* = 0.055	F (2,631) = 67.868, *p* = 0.000

Note: * indicate *p* < 0.05. ** indicate *p* < 0.01.

**Table 13:** Regression analysis of the two dimensions of resilience on fatalism and job stress (N = 639).

Variable	Fatalism		Job Stress	
	Model 7	Model 8	Model 9	Model 10
**Age**	−0.096 (−1.832)	−0.103 (−1.979 *)	0.012 (0.287)	0.007 (0.173)
**Length of Service**	0.030 (0.704)	0.031 (0.733)	0.014 (0.424)	0.014 (0.447)
**Education Level**	−0.088 (−1.897)	−0.070 (−1.533)	0.001 (0.026)	0.014 (0.405)
**Marital Status**	−0.021 (−0.227)	0.009 (0.103)	−0.067 (−0.934)	−0.044 (−0.620)
**Accident Experience**	0.074 (0.681)	0.059 (0.555)	0.263 (3.129 **)	0.252 (3.044 **)
**Tenacity**		−0.241 (−3.150 **)		−0.203 (−3.418 **)
**Strength**		−0.018 (−0.262)		−0.004 (−0.070)
**R^2^**	0.013	0.045	0.021	0.054
**F**	F (5,633) = 1.698, *p* = 0.133	F (7,631) = 4.229, *p* = 0.000	F (5,633) = 2.676, *p* = 0.021	F (7,631) = 5.194, *p* = 0.000
**△R^2^**	0.013	0.032	0.021	0.034
**△F**	F (5,633) = 1.698, *p* = 0.133	F (2,631) = 10.432, *p* = 0.000	F (5,633) = 2.676, *p* = 0.021	F (2,631) = 11.272, *p* = 0.000

Note: * indicate *p* < 0.05. ** indicate *p* < 0.01.

**Table 14:** Regression analysis of resilience on safety behavior (N = 639).

Variable	Safety Behavior	
	Model 1	Model 2
**Age**	0.023 (0.720)	0.021 (0.671)
**Length of Service**	−0.026 (−0.990)	−0.025 (−0.974)
**Education Level**	0.059 (2.087 *)	0.061 (2.205 *)
**Marital Status**	0.039 (0.688)	0.048 (0.872)
**Accident Experience**	−0.189 (−2.841 **)	−0.183 (−2.827 **)
**Safety Attitude**		0.326 (6.111 **)
**R^2^**	0.024	0.078
**F**	F (5,633) = 3.090, *p* = 0.009	F (6,632) = 8.947, *p* = 0.000
**△R^2^**	0.024	0.054
**△F**	F (5,633) = 3.090, *p* = 0.009	F (1,632) = 37.344, *p* = 0.000

Note: * indicate *p* < 0.05. ** indicate *p* < 0.01.

**Table 15:** Regression analysis of the impact of safety attitude dimensions on safety participation and safety compliance (N = 639).

Variable	Safety Compliance		Safety Participation	
	Model 3	Model 4	Model 5	Model 6
**Age**	0.016 (0.628)	−0.009 (−0.469)	0.026 (0.710)	−0.006 (−0.210)
**Length of Service**	−0.015 (−0.709)	0.000 (0.015)	−0.031 (−1.049)	−0.010 (−0.405)
**Education Level**	0.022 (0.966)	0.017 (0.971)	0.081 (2.477 *)	0.075 (2.787 **)
**Marital Status**	−0.013 (−0.292)	0.001 (0.018)	0.074 (1.142)	0.084 (1.586)
**Accident Experience**	−0.089 (−1.700)	0.005 (0.118)	−0.246 (−3.222 **)	−0.093 (−1.466)
**Management Safety Commitment**		0.153 (6.655 **)		0.170 (4.819 **)
**Fatalism**		−0.012 (−0.749)		−0.040 (−1.641)
**Team Safety Climate**		0.333 (12.395 **)		0.451 (10.929 **)
**Job Stress**		0.061 (2.932 **)		−0.046 (−1.435)
**R^2^**	0.008	0.415	0.032	0.361
**F**	F (5,633) = 0.993, *p* = 0.421	F (9,629) = 49.525, *p* = 0.000	F (5,633) = 4.175, *p* = 0.001	F (9,629) = 39.530, *p* = 0.000
**△R^2^**	0.008	0.407	0.032	0.329
**△F**	F (5,633) = 0.993, *p* = 0.421	F (4,629) = 109.339, *p* = 0.000	F (5,633) = 4.175, *p* = 0.001	F (4,629) = 81.082, *p* = 0.000

Note: * indicate *p* < 0.05. ** indicate *p* < 0.01.

**Table 16:** Regression analysis of the mediating role of safety attitude (N = 639).

Variable	Safety Attitude	Safety Behavior	
	Model 1	Model 2	Model 3
**Age**	0.040 (0.452)	0.110 (1.501)	0.104 (1.443)
**Length of Service**	−0.018 (−0.217)	−0.093 (−1.328)	−0.090 (−1.309)
**Education Level**	−0.029 (−0.681)	0.028 (0.783)	0.032 (0.915)
**Marital Status**	−0.047 (−1.135)	−0.033 (−0.951)	−0.026 (−0.763)
**Accident Experience**	−0.009 (−0.217)	−0.090 (−2.712 **)	−0.089 (−2.714 **)
**Resilience**	0.170 (4.268 **)	0.556 (16.775 **)	0.532 (16.037 **)
**Safety Attitude**			0.146 (4.458 **)
**R^2^**	0.029	0.325	0.345
**F**	F (6,632) = 3.163, *p* = 0.005	F (6,632) = 50.617, *p* = 0.000	F (7,631) = 47.521, *p* = 0.000
**△R^2^**	0.028	0.301	0.021
**△F**	F (1,632) = 18.219, *p* = 0.000	F (1,632) = 281.408, *p* = 0.000	F (1,631) = 19.871, *p* = 0.000

Note: ** indicate *p* < 0.01.

**Table 17:** Hierarchical regression of safety attitude mediating the impact of resilience on safety compliance (N = 639).

Variable	Safety Attitude	Safety Compliance	
	Model 1	Model 4	Model 5
**Age**	0.040 (0.452)	0.096 (1.235)	0.083 (1.148)
**Length of Service**	−0.018 (−0.217)	−0.069 (−0.920)	−0.063 (−0.902)
**Education Level**	−0.029 (−0.681)	−0.012 (−0.305)	−0.002 (−0.063)
**Marital Status**	−0.047 (−1.135)	−0.065 (−1.781)	−0.050 (−1.467)
**Accident Experience**	−0.009 (−0.217)	−0.048 (−1.362)	−0.045 (−1.376)
**Resilience**	0.170 (4.268 **)	0.484 (13.716 **)	0.430 (12.862 **)
**Safety Attitude**			0.321 (9.753 **)
**R^2^**	0.029	0.235	0.336
**F**	F (6,632) = 3.163, *p* = 0.005	F (6,632) = 32.429, *p* = 0.000	F (7,631) = 45.524, *p* = 0.000
**△R^2^**	0.028	0.228	0.100
**△F**	F (1,632) =18.219, *p* = 0.000	F (1,632) =188.136, *p* = 0.000	F (1,631) =95.119, *p* = 0.000

Note: ** indicate *p* < 0.01.

**Table 18:** Hierarchical regression of the mediating role of safety attitude between coal miners’ resilience and safety participation (N = 639).

Variable	Safety Attitude	Safety Participation	
	Model 1	Model 6	Model 7
**Age**	0.040 (0.452)	0.107 (1.461)	0.101 (1.400)
**Length of Service**	−0.018 (−0.217)	−0.097 (−1.382)	−0.095 (−1.365)
**Education Level**	−0.029 (−0.681)	0.046 (1.275)	0.050 (1.427)
**Marital Status**	−0.047 (−1.135)	−0.013 (−0.365)	−0.005 (−0.154)
**Accident Experience**	−0.009 (−0.217)	−0.105 (−3.154 **)	−0.104 (−3.167 **)
**Resilience**	0.170 (4.268 **)	0.542 (16.243 **)	0.515 (15.486 **)
**Safety Attitude**			0.158 (4.826 **)
**R^2^**	0.029	0.317	0.341
**F**	F (6,632) = 3.163, *p* = 0.005	F (6,632) = 48.899, *p* = 0.000	F (7,631) = 46.718, *p* = 0.000
**△R^2^**	0.028	0.285	0.024
**△F**	F (1,632) = 18.219, *p* = 0.000	F (1,632) = 263.849, *p* = 0.000	F (1,631) = 23.289, *p* = 0.000

Note: ** indicate *p* < 0.01.

## Data Availability

The study did not report any data.
